# Mind Wandering in Chinese Daily Lives – An Experience Sampling Study

**DOI:** 10.1371/journal.pone.0044423

**Published:** 2012-09-05

**Authors:** Xiaolan Song, Xiao Wang

**Affiliations:** Department of Psychology, Zhejiang Normal University, Jinhua, China; George Mason University/Krasnow Institute for Advanced Study, United States of America

## Abstract

Mind wandering has recently received extensive research because it reveals an important characteristic of our consciousness: conscious experience can arise internally and involuntarily. As the first attempt to examine mind wandering in a non-western population, the present study used experience-sampling method to collect the daily momentary mind wandering episodes in a Chinese sample. The results showed that mind wandering was also a ubiquitous experience among the Chinese population, and, instead of emerging out of nowhere, it was often elicited by external or internal cues. Furthermore, most of the mind wandering episodes involved prospective thinking and were closely related to one’s personal life. Finally, the frequency of mind wandering was influenced by some contextual factors. These results taken together suggest that mind wandering plays an important role in helping people to maintain a continuous feeling of “self” and to prepare them to cope with the upcoming events.

## Introduction

Mind wandering is one of the most ubiquitous experiences in our daily lives. It is generally defined as an involuntary conscious experience that comes from internal mental processes, and is not directly related to the immediate environment or the task at hand [1]–[4]. Mind wandering has received extensive empirical research during the last decade [1], [2], [4]–[7]. The reason for this upsurge of research interest lies in the unique and mystical characteristic of mind wandering: our mind leaves here and now – where it ought to be (the immediate environment or task at hand) – to a remote mental space.

Among the existing studies about mind wandering, one of the most frequently studied topics is the frequency of mind wandering. It has been found that mind wandering, dependent on its context (e.g. during a laboratory task or during a daily life event), occurs 15% to 50% of the time when it is probed [5], [8]–[11]. Another frequently addressed topic is the negative impact of mind wandering on the performance of tasks at hand, it remains controversial whether mind wandering consumes executive resources [4], [12], [13]. The third topic is the relationship between mind wandering and emotions. It has been found that negative emotions would increase the frequency of mind wandering [3], [14], [15], although a recent ESM study proposed that mind wandering is also the cause of unhappy mind [5]. The emotional valence of mind wandering has also been a topic of interest [16], [17]. Finally, the temporal focus of mind wandering, especially its prospective bias, has also received much attention [18]–[20].

However, all the existing studies on mind wandering have been based on data from Western samples [2], [4]–[6]. If mind wandering is indeed a ubiquitous and universal human experience, it should manifest itself in other cultures, not just in the western society. Furthermore, given the strong impact of cultural differences (e.g. the differences between westerners and easterners in thinking style, perception and attention, organization of knowledge) on mental processes [21], [22], the frequency and other characteristics of mind wandering may be different across different cultures. If there exist cross-cultural differences, it should be especially true between the Westerners and Asians, because most of the existing major cultural differences in cognitive processing have been found between Asian and Western individuals.

Regarding the origin and function of mind wandering, a current concern theory has been proposed. It posits that mind wandering comes from the automatic activation of personal goals [23] and serves to direct cognitive resources to the experience of current concerns [4]. One of the major functions of mind wandering thus relates to the anticipation and planning of the future [20]. This theory has received some empirical supports. For example, a significant part of the mind-wandering state during choice reaction time task involves thoughts of the future [24], and a brief period of self-reflection or attention to personal goals increases the prospective bias of the subsequent mind wandering [19], [20]. However, the supportive evidence came only from laboratory studies. To further test this intriguing theory, one needs to test it further in more naturalistic situation, which thus calls for the use of ecological research method. The experience-sampling method (ESM) is one of the most suitable methods for such a purpose.

In the current study, we recruited a large Chinese sample and conducted a detailed examination of the content and context of mind wandering in daily life with the use of the experience-sampling method [25]. In three consecutive days, the participants were probed 6 times a day and completed a questionnaire about their immediate conscious experience each time upon receiving the probe signal. The questionnaire was designed to probe four aspects of mind wandering in daily life: content, context, reasons, and meta-awareness.

## Methods

### 1 Participants

A total of 165 undergraduates volunteered for daily-life experience-sampling through advertisements (115 females), aged from 18 to 29 years (Mean = 20.05 years, *SD* = 1.53). All the volunteers had no history of mental disorder. They were informed of the aim of this study and each one signed an informed consent form. After they returned the questionnaires, they were paid ¥30 for their participation.

The research procedure was in accordance with the ethical principle of the 1964 Declaration of Helsinki (World Medical Organization). The institute review board of Zhejiang Normal University approved the research procedure.

### 2 Materials

The mind wandering questionnaire used in this study was based on a pilot study involving an interview of 20 people, on an examination of the author’s personal diaries, on diary studies about involuntary autographical memory [26] and involuntary semantic memory [27], and on the questionnaire used in another ESM study about mind wandering [9]. The questionnaire contains a total of 23 items. The first item asked participants to judge whether she/he was mind wandering (Q1). The remaining 22 items fell into four categories. The first category was concerned with the contents of mind wandering in terms of episodic or semantic component, including inner speech, visual imagery, auditory imagery, and other sensory imageries (Q3). We define episodic component as any experience of projecting oneself into a simulated scene with spatio-temporal relations and other contextual information, and define semantic component as any thought which is independent of contexts and unrelated to any specific experience. Although visual and auditory imageries are common components in episodic thinking, visual or auditory imagery isolated from its context is generally not classified as an episodic thought and is thus viewed in the present study as a semantic component. The second category was concerned with the reasons of mind wandering, including external or internal cues (Q6), and the extent to which the thought was related to such personal life as one’s self (SELF-R)(Q2), recent experience (RECENT-R)(Q4), and plan (PLAN-R)(Q5). The third category was concerned with the context of mind wandering, including the participants’ current task(s)(Q7), the internal or external orientation of their attention (Q8), their arousal states (Q9), their mood (Q10) and the usage of any psychoactive substance (Q11). The last category was concerned with the meta-awareness of mind wandering, including the awareness of mind wandering and the willingness to let their mind continue to wander once they had been aware of their state of mind wandering (Q12). Q3, Q7, Q10 and Q12 contain several secondary questions. The required responses to all the questions above (including secondary questions) fell into either of the two types. One was making categorical response (the choices varying from item to item), and the other was making the Likert scale from 1, not at all, to 5, very much (except for Q3.4, Q9 and Q10 where scores of 1 to 5 represented the left to the right point of a continuum). The last item of the questionnaire was an open-ended question asking participants to describe in detail what they were thinking about (Q13) (see Questionnaire S1 for the details of the questionnaire).

### 3 Procedure

All the participants received a 30-min training session before the actual study. The research assistants explained the definition of mind wandering. The whole procedure and all the items in the questionnaire were also explained in detail, ensuring that every step of the study procedure and each item were correctly understood. Subsequently, the participants completed one sample questionnaire for practice. They were given a folder containing 18 identical questionnaires. In the following 3 days, the participants were randomly prompted 6 times from 7∶30 a.m. to 11∶30 p.m. per day (twice in the morning, afternoon, and evening, respectively) by the mobile short message (the probe) to fill out a questionnaire. As soon as they received the message, the participants should ponder their conscious experience at that very moment and judge whether she/he was mind wandering. If the answer was “YES,” they should complete all the items in the questionnaire. If the answer was “NO,” they should only answer items 7–11 in the context part of the questionnaire. They were asked to complete the questionnaire within 5 minutes of the probe. If the participants could not complete the questionnaire at that moment they were asked to remember the experience until they have time to do it. After 3 days, the participants were asked to return all the questionnaires in exchange for the subject payment.

## Results

The analysis of the data was conducted for answering the following questions: When, how frequent and why the Chinese minds wander? What occurs to their minds during mind wandering? Is there any impact of the context on their wandering minds? As the proportion of the answer “yes” to Q11 (the use of psychoactive substance) was less then 2%, this item was thus not analyzed.

ESM data have a hierarchical structure in which questionnaire responses (Level 1 data) are nested within participants (Level 2 data), therefore, for each subject, the percentage of each option in the category responses and the mean value of the Likert Scale were calculated, which were used when the descriptive characteristics were reported. Hierarchical linear and nonlinear model (HLM) [28] was used when estimating the relation between the context and mind wandering. We were interested in the generality of the characteristics about mind wandering but not in their individual differences such as gender and age, so no variable was included in Level 2 except participants’ ID.

### 1 How Frequent and When do the Chinese Minds Wander?

The mean rate of mind wandering was 24.4%, a little bit lower than that obtained in previous studies with western participants [5], [9], with considerable variation around the mean (*SD* = 43.0%, range = 100%).

We analyzed whether self-reported mind wandering was systematically associated with particular contexts by HLM. As shown in Table 1, the negative predictors of mind wandering included Q8, Q10.3, Q7.3, and Q7.4, and the positive predictor was Q7.5. That is, participants’ minds wandered less when they were attending to external surroundings, in positive emotion, or concentrated on or good at task(s) at hand, and their minds wandered more when they were doing important task.

**Table 1:** Contextual predictors of the occurrence of mind wandering.

Predictor	Coefficient	*SE*	*t(df)*	*p*
*For all the samples*				
Intercept	−0.988	0.086	−11.451(120)	5.712E-21^***^
Q7: Being on task	0.209	0.126	1.656(120)	0.100
Q8: Attending to external surroundings	−1.176	0.169	−6.941(120)	2.134E-10^***^
Q9: Being at high arousal state	−0.068	0.048	−1.417(120)	0.159
Q10.1: Feeling relaxed	−0.062	0.049	−1.270(120)	0.207
Q10.2: Feeling calm	0.050	0.044	1.150(120)	0.253
Q10.3 Feeling positive emotion	−0.139	0.048	−2.869(120)	0.005**
*For the samples their response to Q7 is “YES”*				
Intercept	−1.223	0.106	11.565(73)	3.613E-18^***^
Q7.1 Challenging task	−0.051	0.056	−0.919(73)	0.362
Q7.2 Interesting task	0.064	0.063	1.010(73)	0.316
Q7.3 Being good at task	−0.214	0.071	−3.011(73)	0.004**
Q7.4 Concentration on task	−0.672	0.084	−8.009(73)	1.354E-11^***^
Q7.5 Important task	0.148	0.068	2.172(73)	0.033*

*Notes:*

1) Only data of 121 participants were included in this analysis because the other 44 participants, after giving a “NO” response to the first question (At the time of the beep, my mind had wandered to something other than what I was doing?), did not answer the items about the context that follow.

2) Samples that made a “YES” response to Q7 (Being on task) were modeled separately with Q7.1–Q7.5 as the predictors.

3) To Q1 and Q7, the answer “YES” was coded as “1” and “NO” was coded as “0”. And the other items used the original codes (same as below);

4) *** *p*<0.001,

** *p*<0.01,

* *p*<0.05 (same as below).

### 2 What do Chinese Think When their Minds Wander?

As the occasions of melody, non-musical sound, and other sensory imagery were rare (Q3), we put them into a new category named as “others”. As shown in Table 2, episodic mind wandering had the highest proportion (60.8%), and was significantly more frequent than other components.

**Table 2:** Components of mind wandering.

	Episodic thought	Inner speech	Visual imagery	Others	*χ^2^/df*	*p*
Mean (%)	60.84	13.95	14.39	10.82	168.07/3***	3.32E-36
*Z*		−8.285***	−8.094***	−9.697***		
*p*		1.18E-16	5.79E-16	3.39E-18		

*Notes:*

1) The Chi-Square value was calculated with the Friedman test for the multiple comparison of the percentage of every component.

2) *Z* value was calculated with the Wilcoxon signed-rank test for the percentage of episodic mind wandering with other components.

3) *N* = 154.

With regard to episodic mind wandering, it has some unique characteristics. We took the temporal orientation as the first unique characteristic of episodic mind wandering (Q3.1), because episodic thought is the only one holding the real experience of “mental time travel”, that is an experience of putting oneself into a scenario that is different from here and now. The result showed that episodic mind wandering was future biased, with the future-oriented episodes (40.53%) being more frequent than other time-orientations (see Table 3). Another unique characteristic of episodic mind wandering was the “Theme”(Q3.2). The proportion of thinking about people(70.95%) was significantly greater than that about objects (29.05%) (*Z = *−5.458, *N = *108, *p = *4.83E-08). The third unique characteristic was the “you-are-there” feeling (Q3.3). The score for “you-are-there” feeling of episodic mind wandering was significantly higher than the median (3, moderately) (see Table 4). The fourth unique characteristic for episodic mind wandering was its emotional valence (Q3.4). The scores of the three dimensions [“Aroused/Relaxed”(Q3.4.1), “Exited/Calm”(Q3.4.2) and “Negative/Positive”(Q3.4.3)] were all significantly higher than the median (3, the mid point of the emotional valence continuum) (see Table 4), suggesting that the individuals tended to think about something relaxing, calming and positive during episodic mind wandering.

**Table 3:** Time orientation of episodic mind wandering.

	Future	Present	Past	No-time orientation	*χ^2^/df*	*p*
Mean (%)	40.53	15.92	21.53	22.02	45.686/3***	6.62E-10
*Z*		−5.266***	−3.924***	−4.023***		
*p*		1.39E-07	8.71E-05	5.74E-05		

*Notes:*

1) The Chi-Square value was calculated with the Friedman test for the multiple comparison of the percentage of every time orientation.

2) *Z* value was calculated with the Wilcoxon signed-rank test for the perception of future oriented episodes with other time orientations.

3) *N* = 142.

**Table 4:** You-are-there feeling and emotional valence of episodic mind wandering.

	You-are-there feeling	Aroused/Relaxed	Excited/Calm	Negative/Positve
Mean	3.38	3.50	3.18	3.32
*t/df*	3.98/136***	6.761/137***	2.372/138***	4.797/137***
*p*	0.0001	3.62E-10	0.0191	4.15E-06

*Notes:*

The table showed the results of the one sample t test for the scores of the Likert Scales nested in episodic option, which were compared with the median 3.

### 3 Are Individuals Aware of their States of Mind Wandering?

In most of the samples (60.11%) the individuals reported that they had found their minds were wandering at the time of the probe (Q12), and the proportion of those with meta-consciousness was higher than those without meta-consciousness (*Z = *−3.193, *N = *154, *p* = 0.001). In the samples of mind wandering with meta-consciousness, more than half of them (55.85%) reported the willingness to continue with it deliberately (Q12.1) (*Z* = −1.747, *N* = 125, *p* = 0.081), which suggests that individuals were not only often aware of their states of mind wandering, but also indulged in it.

### 4 Is there Any Reason for Minds to Wander?

We inferred the reasons of mind wandering from three aspects: the cue of mind wandering, the relation between mind wandering and individuals’ personal lives, and the emotional linkage between the context and the content of episodic mind wandering. In most mind wandering samples (88.17%) the individuals could infer the cue for mind wandering (Q6), the proportion of the samples with cue was significantly more than that of the samples without cue (*Z = *-10.097, *N* = 155, *p = *5.69E-24). Among the samples with cues, nearly a half was induced by internal thoughts (49.43%), which was not different in proportion with the samples induced by external thoughts (*Z = *−0.163, *N = *150, *p = *0.871). The mean scores of SELF-R (Q2) and RECENT-R (Q4) were significantly higher than the median (3, moderately), especially when mind wandering was represented in episodic thinking and inner speech, suggesting a close relation between wandering mind and the individuals’ personal life. In addition, episodic mind wandering was the only one that was significantly considered to be related to personal plan (Q5, PLAN-R) (see Table 5).

**Table 5:** The relation between the context of mind wandering and one’s personal life.

	Episodic thought	Inner speech	Visual imagery	Others	Total
Q2: SELF-R	3.58***	3.59***	3.11	3.06	3.43***
*t(df)*	5.93(133)	3.70(58)	0.58(62)	0.33(46)	5.08(153)
Q4: RECENT-R	3.40***	3.54**	3.03	3.16	3.28**
*t(df)*	4.00(133)	3.01(57)	0.15(62)	0.75(46)	3.32(153)
Q5: PLAN-R	3.20*	3.05	2.57*	2.51*	3.04
*t(df)*	2.00(133)	0.30(57)	−2.15(63)	−2.40(47)	0.59(153)

*Notes:*

The values in the line of Q2, Q4 and Q5 are the mean scores of SELF-R, RECENT-R and PLAN-R respectively. We compared these scores with the median 3.

To explore the emotional linkage between the context and mind wandering, we conducted bivariate correlations between the participants’ mood (Q10) and the content of episodic mind wandering (Q3.4), and found significant positive correlations at all three emotional dimensions between the participants’ mood before the probes and the emotional value of episodic mind wandering (see Table 6). That is, the affective characteristics of episodic mind wandering varied in the same direction with the participants’ emotion.

**Table 6:** The correlation between the participants’ mood before probe and the emotional valence of episodic mind wandering.

Mood before probe	Emotional valence of episodic mind-wandering				
	Q3.4.1 aroused-relaxed	Q3.4.2 excited-calm		Q3.4.3 negative-positive	
Q10.1 aroused-relaxed	.502***		–		–
Q10.2 excited-calm	–		.488***		–
Q10.3 negative-positive	–		–		.634***

*Note:*

The values in the tables are the correlation coefficients.

### 5 The Impact of Context on the Content of Mind Wandering

We analyzed whether the component of mind wandering was systematically associated with particular contexts by HLM. We selected the cues (Q6), the orientation of attention (Q8) and the individuals’ mood (Q10), which were the three factors we were most interested in, as independent variables, and the components of mind wandering (Q3) as dependent variable. As shown in Table 7, the internal cue predicted more episodic mind wandering.

**Table 7:** The impact of context on the content of mind wandering.

Predictor	Coefficient	*SE*	*t(df)*	*p*
*For episodic mind wandering*				
Intercept	2.274	0.197	11.568(139)	4.628E-22^***^
Q6: Cue from internal thoughts	1.004	0.508	1.977(139)	0.050^*^
Q8: Attending to external surroundings	−0.039	0.465	−0.084(139)	0.933
Q10.1: Feeling relaxed	−0.012	0.256	−0.046(139)	0.964
Q10.2: Feeling calm	−0.263	0.228	−1.151(139)	0.252
Q10.3 Feeling positive emotion	0.225	0.196	1.148(139)	0.253
*For inner speech*				
Intercept	0.659	0.238	2.766(139)	0.007^**^
Q6: Cue from internal thoughts	0.855	0.628	1.361(139)	0.176
Q8: Attending to external surroundings	0.110	0.576	0.191(139)	0.849
Q10.1: Feeling relaxed	−0.136	0.257	−0.528(139)	0.598
Q10.2: Feeling calm	−0.287	0.259	−1.110(139)	0.269
Q10.3 Feeling positive emotion	−0.061	0.203	−0.300(139)	0.765
*For visual imagery*				
Intercept	0.439	0.228	1.929(139)	0.055
Q6: Cue from internal thoughts	0.884	0.559	1.582(139)	0.116
Q8: Attending to external surroundings	0.553	0.617	0.897 (139)	0.372
Q10.1: Feeling relaxed	0.308	0.265	1.163(139)	0.247
Q10.2: Feeling calm	−0.289	0.254	−1.140(139)	0.257
Q10.3 Feeling positive emotion	−0.015	0.209	−0.070(139)	0.945

*Notes:*

1) The outcome variable “the components of mind wandering (Q3)” was multinominal, so three models were estimated separately for episodic thought, inner speech, and visual imagery, and the fourth category “others” was the reference category.

2) The trichotomus response to Q6 was coded by two dummy variables:”Cue from internal thoughts” and “ Cue from external surroundings”, and only “Cue from internal thoughts” was entered into the multilevel model because these two variables were negatively correlated.

### 6 The Impact of Context on the Relation between Mind Wandering and Personal Life

Three HLMs were conducted to explore the impact of context on the relation to personal life of mind wandering. The dependent variable was SELF-R, RECENT-R and PLAN-R respectively. As shown in Table 8, internal cue, being good at task and feeling positive emotion were positive predictors of SELF-R. As to RECENT-R, concentration on task was the positive predictor, and interesting task was the negative predictor. And as to PLAN-R, the internal cue and feeling positive emotion were the positive predictors, and feeling calm emotion was the negative predictor.

**Table 8:** The impact of context on the relation to personal life of mind wandering.

Predictor	Coefficient	*SE*	*t(df)*	*p*
*When the predicted is SELF-R* *For all the samples*				
Intercept	3.589	0.078	46.284(141)	3.825E-87^***^
Q6: Cue from internal thoughts	0.510	0.174	2.935(141)	0.004^**^
Q7: Being on task	−0.010	0.163	−0.060(141)	0.952
Q8: Attending to external surroundings	−0.159	0.152	−1.047(141)	0.297
Q9: Being at high arousal state	−0.008	0.077	−0.103(141)	0.918
Q10.1: Feeling relaxed	−0.145	0.076	−1.901(141)	0.059
Q10.2: Feeling calm	−0.089	0.075	1.187(141)	0.238
Q10.3 Feeling positive emotion	0.178	0.075	2.391(141)	0.018^*^
*For the samples their response to Q7 is “YES”*				
Intercept	3.501	0.118	29.760(80)	4.989E-45^***^
Q7.1 Challenging task	0.024	0.078	0.307(80)	0.759
Q7.2 Interesting task	−0.201	0.105	−1.908(80)	−0.060
Q7.3 Being good at task	0.389	0.122	3.201(80)	0.002^**^
Q7.4 Concentration on task	0.071	0.084	−0.845(80)	0.401
Q7.5 Important task	0.008	0.093	0.091(80)	0.928
*When the predicted is RECENT-R* *For all the samples*				
Intercept	3.441	0.084	40.968(141)	3.461E-80^***^
Q6: Cue from internal thoughts	0.006	0.144	0.044(141)	0.965
Q7: Being on task	−0.085	.165	−0.515(141)	0.607
Q8: Attending to external surroundings	−0.214	0.166	−1.292(141)	0.199
Q9: Being at high arousal state	0.030	0.067	0.446(141)	0.656
Q10.1: Feeling relaxed	−0.154	0.089	−1.730(141)	0.085
Q10.2: Feeling calm	−0.121	0.085	−1.415(141)	0.157
Q10.3 Feeling positive emotion	−0.056	0.756	−0.747(141)	0.456
*For the samples their response to Q7 is “YES”*				
Intercept	3.604	0.116	31.143(80)	1.753E-46^***^
Q7.1 Challenging task	0.022	.073	0.299(80)	0.766
Q7.2 Interesting task	−0.327	0.107	−3.045(80)	0.004**
Q7.3 Being good at task	0.195	0.109	1.796(80)	0.076
Q7.4 Concentration on task	0.262	0.101	2.581(80)	0.012*
Q7.5 Important task	−0.004	0.094	0.047(80)	0.963
*When the predicted is PLAN-R* *For all the samples*				
Intercept	3.127	0.090	34.825(141)	3.944E-71^***^
Q6: Cue from internal thoughts	0.713	0.176	4.044(141)	8.613E-05^***^
Q7: Being on task	−0.022	0.167	−0.135(141)	0.894
Q8: Attending to external surroundings	−0.124	0.167	−0.740(141)	0.460
Q9: Being at high arousal state	0.066	0.077	0.857 (141)	0.393
Q10.1: Feeling relaxed	−0.144	0.080	−1.792(141)	0.075
Q10.2: Feeling calm	−0.151	0.075	−2.002(141)	0.047^*^
Q10.3 Feeling positive emotion	0.194	0.080	2.432(141)	0.016^*^
*For the samples their response to Q7 is “YES”*				
Intercept	3.207	0.135	23.705(80)	4.866E-51^***^
Q7.1 Challenging task	−0.070	0.105	−0.665(80)	0.508
Q7.2 Interesting task	0.070	0.128	−0.550(80)	0.583
Q7.3 Being good at task	0.022	0.153	0.144(80)	0.886
Q7.4 Concentration on task	−0.044	0.130	−0.338(80)	0.736
Q7.5 Important task	0.184	0.114	1.618(80)	0.109

Notes:

Three models were estimated separately for SELF-R, RECENT-R and PLAN-R;

Samples that made a “YES” response to Q7 (Being on task) were modeled separately with Q7.1-Q7.5 as the predictors.

The trichotomus response to Q6 was coded by two dummy variables:”Cue from internal thoughts” and “ Cue from external surroundings”, and only “Cue from internal thoughts” was entered into the multilevel model because these two variables were negatively correlated.

## Discussion

In the current study we conducted a detailed examination of the day-to-day mind wandering experiences through the experience sampling method. To the best of our knowledge, the present study is the first investigation of mind wandering in a population other than Westerners. We collected a host of information regarding the content, context, reasons and meta-awareness of mind wandering as well as its frequency. We found that mind wandering was also a ubiquitous and continuous experience among the Chinese population. We also found mind wandering was often elicited by external or internal cues rather than emerging out of nowhere. Furthermore, most of the mind wandering episodes involved prospective mental time travel and were closely related to one’s personal life. Finally, the frequency of mind wandering was influenced by a number of factors, such as attention orientation, devotion to task, and mood. These results taken together suggest mind wandering plays an important role in helping people to maintain a continuous feeling of “self” and to prepare them to cope with the upcoming events.

The current study replicated several previous observations made by prior works. First, we confirmed that a significant proportion of daily cognition was made up by thoughts unrelated to here and now. In our sample, the frequency of mind wandering was 24.4%, which was within the range of those obtained in the past studies from the laboratory [4] and the day-to-day living [5], [9]. Second, the frequency of mind wandering was influenced by some contextual factors. The individuals’ minds wandered less during concentration or when they were doing what they were good at, but wandered more when they were doing important tasks or in negative mood. These findings are consistent with previous studies: minds wandered less when participants were concentrated or felt competent [9], while the decreased demand of a task [6], [29], stressful task and negative mood increased its frequency [3], [15]. Third, the result that external attention decreased the frequency of mind wandering supports the hypothesis that mind wandering represents the decoupling of attention resources from the sensory information to the internal train of thoughts [30], as the mind is more decoupled from the current environment when individuals pay their attention to the internal world than when they pay their attention to the external world. Fourth, the mind wandering episodes were mainly episodic in nature. This result is consistent with the findings from the studies on involuntary memory which found the episodic involuntary memory to be more frequent than the semantic one [27], [31], and with a recent study that found visual mental imagery (which included episodic representation in that study) to be the predominant of the participants’ inner experiences during the resting state [32]. Moreover, the inner speech or inner language was found to be one of the main types of inner experience in the current survey and some other studies [32], [33].

These results extend our understanding of several specific characteristic features of mind wandering. First, when it comes to the predominant episodic component, mind wandering is mainly a kind of mental time travel, and its prospective-bias may be an important trait of the healthy population. This prospective-bias has been verified in several recent laboratory studies [19], [20], [24] and the content and function of future-oriented thoughts in daily life has been investigated by a recent experience sampling study [17].

However, our findings go beyond the previous works in several ways. Our results were more confined to the temporal orientation in episodic mind wandering because only during this type of episodes participants could have the real experience of mental time travel, that is, projecting oneself into an alternative situation [34]. The significant “you-are-there” feeling of episodic mind wandering suggests a state of autonoetic consciousness [35], [36]. Mind wandering in semantic form does not have this experience although its contents can be linked to past or future events too. We propose that this prospective bias in involuntary mental time travel represents a very important adaptive function of mind wandering. We further found that when being induced by internal thoughts, the episodic bias was strengthened, accompanied by higher self and plan relevance. Our result is consistent with Smallwood and his colleagues who concluded that self-reflection is a core component of future thinking during mind wandering [19]. Based on these results, we suggest that mind wandering plays a relevant role in the formation of self-consciousness. It is one’s past and future that make the content of the mental “self”, and mind wandering provides us the platform (but not the only platform) on which we experience ourselves. When we do internal thinking the mental self is more activated and the mind wandering with autonoetic consciousness is more vigorous. Consequently, the internal thoughts induce more episodic mind wandering and lead the spontaneous episodes to be strongly associated with one’s self and plan.

We further found that mind wandering is not a random conscious experience. Both external and internal cues could induce the mind to wander, and what the participants thought during mind wandering was linked closely to their personal lives, such as the recent experience and personal plan which were closely related to the “self”. These findings can help to explain the causes of mind wandering, which are embodied in two aspects–one at a conscious level and the other at an unconscious level. At the conscious level, mind wandering could be triggered involuntarily by cues in the external environment or by our own mind. In most cases the participants could determine the origin of the immediate mind wandering experience, which came from external surroundings or internal thoughts. At the unconscious level, the close relation between the content of mind wandering and one’s personal life suggests the potential motivation of mind wandering. Its tight relation to one’s self and recent or future life, especially in the episodic form, indicates that what we think during mind wandering belongs to the internal personal goal frame or current concerns [12], [37], [38]. These internal mental events could be processed unceasingly in an unconscious manner; once the executive control for the task weakens [12], the external and internal cues would trigger the internal mental events to go into the conscious global workspace [39].

Furthermore, the degree to which mind wandering is related to one’s personal life was influenced by the immediate context. The results showed that internal cue and positive emotion predicted a closer relation of mind wandering to one’s self and plan, and calm emotion predicted a lower relevance with one’s plan (that is, excited emotion predicted a higher relevance between mind wandering and one’s plan). One possible reason is that internal cue induced more episodic mind wandering which has higher relevance with personal life, and another possibility is related to the prospective function of mind-wandering [19]. It is understandable that the cue came from one’s internal mental world made one’s wandering mind more related to one’s self, because the cue itself came from the individual’s self, and planning of the future is one function of self. Positive or exited emotion made the planning function of mind wandering more salient, which was consistent with the finding that unhappy moods lead to a retrospective rather than a prospective bias to mind wandering [40]. On the other hand, it is argued that positive emotion can broaden attention [41], [42], and more available attention resources could strengthen the prospection of mind wandering [18]. We believe that more available attention resources will make the content of mind wandering more “reasonable”, that is, more related to one’s personal life. Consistence with this, interesting or demanding tasks decreased this reasonability of mind wandering. That is, when individuals were doing interesting tasks their wandering minds had the lower RECENT-R, and when individuals were doing the tasks that they were good at (less demanding), their wandering minds had a higher SELF-R.

Surprisingly, concentration and the interestingness of task showed contrary effects on the RECENT-R of mind wandering. That is, contrary to the interestingness of task, being concentrated on task was the positive predictor of RECENT-R. This result seemed inexplicable since both being concentrated on and interested in task occupy more attention resources. One possible reason is that concentration on task can increase the emotional arousal. Therefor like the excited emotion, it increased the relation between mind wandering and one’s personal life; but unlike the excited emotion, the effect of concentration was manifested in the wandering mind’s relevance with recent experience rather than plan. Generally, in the present study the exact effect of the individuals’ emotion on the relevance with personal life of mind wandering is not clear and needs to be studied intensively in future. Taken together, the results about the relation of mind wandering and personal life suggest that mind wandering is not the mind’s random noise but rather it is both functional and adaptive.

The emotional linkage between the participants’ mood and mind wandering is also worth noting, which suggests mind wandering’s close relation with reality, as has been reported by other studies of involuntary autobiographical memory [26]. It also coincides with our experience that when we feel happy, we tend to think of happy things and when we feel depressed, negative thoughts always come unasked. This kind of relation makes one’s experience and emotion coherent, and can prevent the emotion from fluctuating excessively. Thus, mind wandering may also play a role in emotional regulation. However, the latest survey of mind wandering has demonstrated that it is the wandering mind that makes us unhappy, but not vice versa [5], which is somewhat inconsistent with the previous conclusions that negative emotions make the mind more likely wander off [15] and our findings that negative emotion was the predictor of mind wandering. Future experimental researches are thus still needed to clarify the relation between emotion and mind wandering.

An unexpected result is that the participants were often aware of their states of mind wandering and allowed the states to go on. A previous research found that the frequency of mind wandering measured by the probe-caught method was higher than the one measured by the self-caught method [4], [43], which suggests that people often know less about the fact that their minds have wandered. Our results suggest that mind wandering in daily life may be more self-conscious and voluntary than that indicated by laboratory studies. Combined with the result of our pilot study, in which 20 participants were interviewed about their mind wandering in daily life and the interviewees manifested great acceptance for mind wandering, the high rate of tolerance with mind wandering suggests that individuals treat mind wandering as an ordinary phenomenon and make peace with it, and even like it.

One of the most important contributions of the present study is that we extended the current work on mind wandering to a population other than western people. Here the participants from China expressed a similar tendency of mind wandering, and the relative large individual difference in frequency is in accordance with the study conducted in the West [9]. However, the frequency of mind wandering in our sample is a little bit lower than those existing studies [5], [9]. One possible reason may lie in the relatively strict criterion of mind wandering in our study. However, we could not exclude the possibility that Chinese minds do wander less than American and European ones given the large differences between two cultures. For example, contrast to western cultures, eastern Asian cultures value more serious and ordered thinking style. On the other hand, there are substantial similarities in mind wandering between Chinese and Western populations, such as the prospective-bias of mind wandering [19], [20], and the close relation between negative mood and mind wandering [3], [15]. These similarities among different cultures suggest that some adaptive functions of mind wandering may be universal and culture-independent. Having said that, considering the dramatic differences in the nature of Asian and European thought processes [22], we strongly recommend cross-cultural research on mind wandering in the future such that culture-dependent and culture-independent characteristics of mind wandering could be elucidated.

### Conclusions

The present study, using the ESM, collected the daily experiences with a large Chinese sample. We found that as it is the case with Westerners, mind wandering was a ubiquitous and continuous experience among the Chinese population. We also found mind wandering was often elicited by external or internal cues instead of emerging out of nowhere. Furthermore, most of the mind wandering episodes involved prospective mental time travel and were closely related to one’s personal life. The frequency of mind wandering was influenced by a number of factors, such as attention orientation, devotion to task, and mood. These results taken together suggest that mind wandering plays an important role in helping people to maintain a continuous feeling of “self” and to prepare them to cope with the upcoming events.

## Supporting Information

Questionnaire S1 **The Mind wandering Questionnaire.**
(DOC)
