# Supplementary material for: Mind Wandering in Chinese Daily Lives – An Experience Sampling Study
Source: PLoS One. 2012 Sep 5;7(9):e44423. doi: 10.1371/journal.pone.0044423 (PMC3434139; doi:10.1371/journal.pone.0044423)
Supplement: Questionnaire S1 — The Mind wandering Questionnaire. (DOC) [file pone.0044423.s001.doc]

**The Mind wandering Questionnaire**

The Likert scale ranges from not at all (1) to very much (5) except questions 2.4, 8, and 9 in which scores of 1 to 5 represented the left to the right point of a continuum.

| Q1 At the time of the beep, my mind had wandered to something other than what I was doing?  ①Yes ②No  *(If the answer is “*②*No”, please go directly ahead to Q 7-11 and leave out the others.)* |
| --- |
| Q2 My wandering thought was related to myself. 1 2 3 4 5 |
| Q3 My wandering thought was  ①episodic thought ②inner speech ③visual image ④melody ⑤unmusical sounds ⑥other sensory imagery  *(If the answer is “①episodic thought”, then answer Q3.1-3.4, otherwise please go directly ahead to Q 4)*  If my wandering thought was episodic, then  Q 3.1 My wandering thought was   1. about past events ②about present events ③about future events   ④no temporal orientation  Q 3.2 My wandering thought was about ①people ②objects  Q 3.3 When my mind was wandering I had a “you-are-there” feeling.  1 2 3 4 5  Q 3.4 The episodes in my mind make me feel  3.4.1 aroused---relaxed 1 2 3 4 5  3.4.2 excited---calm 1 2 3 4 5  3.4.3 sad(negative)---happy(positive) 1 2 3 4 5 |
| Q4 My wandering thought was related to my recent life experience.  1 2 3 4 5 |
| Q5 My wandering thought was related to my plan. 1 2 3 4 5 |
| Q6 The cue of my wandering thought was from  ①external surroundings ②my own internal thoughts ③no reason |
| Q7 I was doing some task at the time of the signal. ①Yes ②No  *If the answer is “①Yes”, then answerQ 7.1-7.5, otherwise please go directly ahead to Q 8*  7.1 What I was doing just now is challenging. 1 2 3 4 5  7.2 I’ was interested in what I was doing just now. 1 2 3 4 5  7.3 I’m good at what I was doing just now. 1 2 3 4 5  7.4 I was concentrated on what I was doing just now. 1 2 3 4 5  7.5 What I’ was doing just now is important to me. 1 2 3 4 5 |
| Q8 Prior to the signal, I was attending to :  ①internal thoughts ②external surroundings |
| Q9 Prior to the signal, my arousal state was: Sleepy------lucid  1 2 3 4 5 |
| Q10 Prior to the signal, I felt :  Q10.1 aroused------relaxed 1 2 3 4 5  Q10.2 excited-------calm 1 2 3 4 5  Q10.3 sad(negative) ------ happy(positive) 1 2 3 4 5 |
| Q11 I have drunk wine or coffee, smoked or taken drugs today. ①Yes ②No |
| Q12 By the time of the beep I had known that my mind was wandering  ①Yes ②No  If the answer is “Yes”, please answer Q12.1, otherwise go directly ahead to Q13.  Q12.1 I had realized my mind was wandering but still let my mind continue to wander deliberately.  ①Yes ②No |
| Q13 Please give a detailed description of your wandering thought (You are free to choose to answer it or keep it a secret). |
